# The social gradient in work and health: a cross-sectional study exploring the relationship between working conditions and health inequalities

**DOI:** 10.1186/1471-2458-13-1170

**Published:** 2013-12-13

**Authors:** Oliver Hämmig, Georg F Bauer

**Affiliations:** 1 Division of Public and Organizational Health, Institute of Social and Preventive Medicine, University of Zurich, Hirschengraben 84, 8001, Zurich, Switzerland; 2 Center for Organizational and Occupational Sciences, Swiss Federal Institute of Technology Zurich (ETH Zurich), Weinbergstr. 56/58, 8092, Zurich, Switzerland

**Keywords:** Health inequalities, Social gradient, Educational status, Occupational status, Physical working conditions, Psychosocial working conditions, Blue-collar job characteristics, White-collar job characteristics, Switzerland

## Abstract

**Background:**

Social inequalities in health are widely examined. But the reasons behind this phenomenon still remain unclear in parts. It is undisputed that the work environment plays a crucial role in this regard. However, the contribution of psychosocial factors at work is unclear and inconsistent, and most studies are limited with regard to work factors and health outcomes. This study, therefore, aimed to explore the role and contribution of various physical and psychosocial working conditions to explaining social inequalities in different self-reported health outcomes.

**Methods:**

Data from a postal survey among the workforces of four medium-sized and large companies from diverse industries of the secondary sector in Switzerland were used and analysed. The study sample covered 1,846 employees aged 20 and 64 and included significant proportions of unskilled manual workers and highly qualified non-manual workers. Cross tabulations and logistic regression analyses were performed to study multiple associations between social status, work factors and health outcomes. Combinations of educational level and occupational position wee used as a measure of social status or class.

**Results:**

Clear social gradients were observed for almost all adverse working conditions and poor health outcomes studied, but in different directions. While physical workloads and other typical blue-collar job characteristics not suprisingly, were found to be much more common among the lower classes, most psychosocial work demands and job resources were more prevalent in the higher classes. Furthermore, workers in lower classes, i.e. with lower educational and occupational status, were more likely to report poor self-rated health, limited physical functioning and long sickness absence, but at the same time were less likely to experience increased stress feelings and burnout symptoms showing a reversed health gradient. Finally, blue-collar job characteristics contributed substantially to the social gradient found in general and physical health outcomes. In contrast, white-collar job characteristics made no contribution to explaining the gradient in these health outcomes, but instead largely explained the reversed social gradient observed for the mental health outcomes.

**Conclusion:**

The findings suggest a more differentiated pattern of the commonly found social gradient in health and the differential role of work in this respect.

## Background

Social inequalities in health are well documented in the literature and have been demonstrated for a number of general health outcomes and chronic diseases in a large number of studies from a broad range of countries. Numerous international studies and mortality statistics have revealed wide health inequalities within and between social classes and countries [1-5]. There is overwhelming evidence particularly but not only from the famous Whitehall studies showing that morbidity and mortality rise steadily with gradually decreasing social or socioeconomic status [4-7]. This inverse and graded relationship in individuals is consistently observed both with educational and occupational status [4] and is commonly known as the social gradient in health and disease, also referred to as the status syndrome [8,9]. This fundamental association runs across society [7], has occurred and been observed at all times, is found in almost every industrialized nation in which it has been studied [2], and therefore is considered to be an almost universal phenomenon. And although health in general has improved, morbidity and mortality rates have significantly and continuously declined and overall life expectancy has remarkably and steadily increased over the past decades, social inequalities in health, disease and life expectancy have widened rather than declined in modern affluent societies [4,10-14].

### Explaining the gradient

The reasons, mechanisms and pathways behind this graded association and inverse relation between social status (or socioeconomic position) and health are not yet fully understood [15,16]. Several possible explanations have been provided and studied [1,11,15]. Particular attention was directed early on to critical and stressful life events, to lifestyle factors and health risk behaviours, to the healthcare system, to socialization and the social environment, to the living and housing environment and exposure to environmental hazards. However, these factors and conventional explanations only partly explain the social gradient in health [9,17].

Another important explanation that has received some scientific attention in recent years is the role and contribution of the work environment [7,10,11,15,17-22]. Since work stress and unemployment have long been recognised as important social determinants of health [7,14], and a number of work demands, job characteristics and occupational exposures have been found to be associated with socioeconomic or occupational status [11,16], the work environment is considered to be one of the major sources of social inequalities in health [10,16,22]. The psychosocial work environment has been particularly studied in this regard [10,11,20,23-25]. But physical job demands and exposures have also been found to contribute to social inequalities in health [10,15,16,19,26]. While there is broad and consistent evidence that physical working conditions show clear social gradients and clearly mediate the relationship between social status and health, the gradients and contributions or effects of psychological work demands or psychosocial work factors are much less clear and uniform [10,15,16,19,25,26].

### Limitations and focuses of previous studies

As regards the work environment and its contribution to the social gradient in health, most recent studies have focused either on isolated or a limited number of mainly psychosocial work factors or on individual health outcomes, and their findings have produced only a fragmented picture. Psychosocial work factors that have been studied and found to partly explain social inequalities in health are largely restricted to job control or decision authority, skill discretion, social support at work and job insecurity [20,22-24,27]. And the contribution of psychosocial work factors to the social gradient in health has been studied predominantly in relation to cardiovascular disease and mortality [20,23,27-29] and/or self-rated health [10,17,18,21,26].

Only few previous studies have simultaneously examined various work factors, and particularly the contribution of combined physical *and* psychosocial working conditions to explaining the social gradient in health and disease [16-19,26,27,30]. Furthermore, only a handful of studies have looked at different general, physical and/or mental health outcomes other than or in addition to self-rated health or cardiovascular disease [16,17,24,25,30,31]. And, finally, many studies with some exceptions [10,16,26,29] have relied on middle-class cohorts or large population samples that are exclusively composed of, or at least strongly dominated by, well-educated employees in non-manual jobs and high-status occupations (white-collar workers). So unskilled workers in manual and low-status jobs (blue-collar workers) have typically been either excluded or strongly underrepresented in study samples [4,5,28].

A previous study that we carried out on the social gradient in health and the role of work in this relationship used nationally representative data from the Swiss Health Survey, but was nevertheless focused on self-rated health as the only health outcome studied, and was limited to just those working conditions available in the Swiss Health Survey [32].

### Study aims and research questions

In view of these limitations and shortcomings of the research literature, it has been suggested that a study should cover the whole range of workers and social classes and all types of work factors and occupational exposures [16] that may be of health relevance. Accordingly, the present study includes various physical *and* psychosocial working conditions, considers several health outcomes aside from self-reported health, and covers the full range of social status groups from university graduates in management positions and other highly educated employees in higher occupational positions to poorly qualified manual workers without supervisory functions [16,17].

The main aim of the study was to investigate the contribution of a wide range of physical and psychosocial work factors to explaining the social gradient in health and to determine whether or not these job strains and stressors play a differential and conflicting role and possibly cancel each other out in this respect. Secondary objectives were (a) to study associations of these work factors with social status or class, (b) to examine whether these adverse working conditions turn out to be health risk factors, and finally (c) to explore possible variations in strength and/or direction of the social gradient in health across various general, physical and mental health outcomes.

Following previous studies [15,17,18,27,30], we expected less educated employees in low-status and blue-collar jobs to have fewer work-related resources and rewards and to face greater physical demands and dangerous exposures at work. In contrast, we expected higher educated employees with white-collar jobs and high job status to enjoy greater resources and rewards, but also to be more likely to experience psychosocial demands at work [10,15,16,18,25]. Consequently, we expected physical work demands and other typical blue-collar job characteristics to partly or fully explain the social gradient in health, and at least some white-collar job characteristics and psychosocial work demands to rather mask this gradient.

## Methods

### Data and study sample

For the present study, we used cross-sectional data from a postal employee survey which was conducted in spring 2010 in the industry sector in Switzerland. Data were collected by full surveys among the workforces of four Swiss companies of varying size and from different regions and diverse industries (metal working, pharmaceuticals and chemicals, mechanical engineering and construction). A total of 2,014 written questionnaires had been completed and returned, but statistical analyses were restricted to 1,846 employees between 20 and 64 years of age, excluding employees aged 16 to 19 and mostly still in education (apprentices, trainees).

The business locations or head offices of the participating companies had between 200 and some 2,800 employees at the time of data collection. The overall participation or return rate (RR) was slightly over 49%. The participating companies were a) a well-known producer of cutlery, watches and pocket knives located in Central Switzerland (n = 397, RR = 49.0%), b) a multinational developer and manufacturer of pharmaceuticals and agrochemicals and a leading supplier to the pharmaceutical, healthcare and life science industries from south-western Switzerland (n = 1358, RR = 48.2%), c) a leading producer, distributor and service provider in the printing and labelling machine industry from eastern Switzerland (n = 171, RR = 66.3%), and finally d) a comparatively large construction company in the Zurich area (n = 88, RR = 44.2%).

The heterogeneous study sample included employees from all educational levels and occupational positions, the full range from unskilled construction and industrial workers in production positions (‘blue-collar workers’) to highly qualified employees in supervisory or management positions (‘white-collar workers’). Men, full-time employees, older and less educated or unskilled workers were slightly or strongly overrepresented in the study population compared to a nationally representative and weighted sample of the employed population in Switzerland in the same age range (see Table 1). This simply reflects the diverse workforce demographics in the industry sector compared to the dominant service sector in Switzerland. As usual and could have been expected among low skilled blue-collar workers, foreign employees were less likely to participate in the study than their Swiss colleagues due to language difficulties and understanding problems, and therefore underrepresented in the study sample.

**Table 1:** Characteristics of the study population stratified by sex and in comparison with a nationally representative standard population of employees in Switzerland

		**Study population**			**Standard population**^**a**^
		**Employee survey 2010 in the industry sector (N = 1,846)**			
		**Men**	**Women**	**Total**	
**Sex**	Men	100.0%	–	81.8%	53.0%
	Women	–	100.0%	18.2%	47.0%
**Age**	20-25 years	6.3%	11.3%	7.2%	11.2%
	26-35 years	16.4%	25.0%	17.9%	22.9%
	36-45 years	30.1%	33.9%	30.8%	27.7%
	46-55 years	31.9%	20.6%	29.9%	24.9%
	56-64 years	15.3%	9.2%	14.2%	13.3%
**Education** (highest level achieved)	No compulsory or vocational education	20.0%	33.9%	22.5%	14.5%
	Basic vocational education	48.8%	42.7%	47.7%	42.6%
	Higher vocational education	22.3%	12.4%	20.5%	24.5%
	University degree	8.9%	10.9%	9.3%	18.4%
**Nationality**	Swiss (incl. dual citizenship)	88.2%	76.8%	86.1%	77.1%
	Other nationality	11.8%	23.2%	13.9%	22.9%
**Job status** (occupational position)	Management position (directorate)	0.9%	0.0%	0.8%	5.0%
	Supervisory position (executive staff)	34.1%	12.2%	30.2%	26.8%
	Production position (regular staff)	64.9%	87.8%	70.0%	68.2%
**Activity rate**	Part-time (< 100%)	4.1%	27.7%	8.4%	41.6%
	Full-time (100%)	95.9%	72.3%	91.6%	58.4%

^a^ Based on data of the Swiss Household Panel 2009 (collected between September 2009 and March 2010) and a weighted random sample of the resident population in Switzerland restricted to employees aged 20 to 64 (N=3,714).

The study is observational and not clinical or experimental and did not involve drugs, medical records or human tissues. Survey data were collected on a voluntary and anonymous basis, and in particular not from hospitals (patients), retirement homes (pensioners) or prisons (prisoners). Therefore no approval was required by the ethics committee nor any authorisation by the commissioner for data protection by the national and cantonal laws nor were these recommended by the medical-ethical guidelines for scientific integrity of the Central Ethics Committee and the Swiss Academies of Sciences.

### Measures

#### Social class

In this study, social status or class was measured by two indicators, namely educational level and occupational position. Educational status was originally categorized on a 10-point ordinal scale and then consolidated to four educational levels (see Table 1). Occupational status was classified along a three-level hierarchy (see Table 1). As status consistency and the association between educational and occupational status was fairly high (Gamma coefficient = .67), a combined variable was created and used as a measure of social class with five different hierarchically structured combinations of educational level and occupational position (see Table 2). Wage or household income was not surveyed at all given the many unreliable or refused answers and missing values that usually come along with delicate questions like these, particularly in Switzerland.

**Table 2:** Association of occupational position and educational level (with categories of social class)

	**No or only compulsory education (n = 408)**	**Basic vocational education (n = 869)**	**Higher vocational education (n = 373)**	**University degree, doctorate (n = 169)**	**Total (N = 1,819)**
**Production position** (n = 1,256)	89.2% ^a^	78.5% ^b^	45.3% ^c^	24.3% ^d^	**69.0%**
**Supervisory position** (n = 549)	10.8% ^b^	21.4% ^c^	53.1% ^d^	71.6% ^e^	**30.2%**
**Management position** (n = 14)	0.0% ^c^	0.1% ^d^	1.6% ^e^	4.1% ^e^	**0.8%**
**Total** (N = 1,819)	**100.0%**	**100.0%**	**100.0%**	**100.0%**	**100.0%**

Gamma coefficient = 0.67, p < .001.

^a^ class I (n = 364), ^b^ class II (n = 726), ^c^ class III (n = 355), ^d^ class IV (n = 240), ^e^ class V (n = 134).

#### Physical and psychosocial work factors

Physical work factors were measured by asking directly about physical demands and strains as well as adverse ergonomic exposures at work. A total of five single items or questions about carrying or moving heavy loads, uniform arm or hand movements, painful or tiring posture, repetitive work, and having a strenuous job were used as measures of physical working conditions, mainly taken from the Swiss Health Survey. Participants were asked either with a yes/no question or on a 5-point scale (from “fully” to “not at all”) if or to what degree such work demands and characteristics applied to their job. Demands and exposures that were reported to apply “fully”, “largely” or “partly” to one’s work situation were considered to be characteristic of their job.

Psychosocial work factors covered various psychosocial demands (e.g. high time pressure, frequent interruptions, growing workload, a lot of responsibility, status inconsistency, monotonous work, poor work-life compatibility, regular overtime, regular work time changes at short notice). But they also included limited resources or unavailable rewards such as low job autonomy, inflexibility of working hours, job insecurity, low social support, and poor promotion prospects or inappropriate career opportunities. With the exception of job autonomy, these psychosocial work factors were measured by single items mostly selected from the established Effort-Reward Imbalance (ERI) Questionnaire of Siegrist et al. [33]. Job insecurity, for example, was assessed by a simple yes/no question about the risk of job loss (“My job security is poor.”) in accordance with the ERI questionnaire. Social support at work was likewise measured by a single item from the ERI questionnaire (“I experience adequate support in difficult situations.”). Work-life compatibility was again assessed by a single item (“Do your working hours fit in with your family or social commitments outside work?”) from the European Working Conditions Survey [34]. And flexibility of working hours was measured by a multiple response question about one’s work schedule or working time arrangement. Having fixed working hours or working in shifts, at weekend, at night, on call or being on standby without simultaneously indicating flexible working hours was categorized as having inflexible working hours. In contrast to these single-item measures, job autonomy was measured using 14 items taken from two validated multiple-item scales on the influence (10 items) and degree of freedom (4 items) at work of the long research version of the Copenhagen Psychosocial Questionnaire of Kristensen, Hannerz, et al. [35]. These 5-point scaled items (with scores from 0 ‘never’ to 4 ‘always’) were added up to a multiple-item measure with a summarized score ranging from 0 to 56 (Cronbach’s alpha = .80). A total score of 26 or below was considered to be indicative of a comparably low degree of job autonomy. In total, 14 items and scales were used as measures of psychosocial work demands and resources.

#### General, physical and mental health outcomes

Self-rated health and self-reported absence from work due to sickness were used as global health measures. Self-rated health, a 5-point Likert-scaled single item, is the most commonly used and probably the best validated general health indicator used in health surveys and population studies, also known and confirmed as a good predictor of overall morbidity and mortality [36]. Respondents who rated their general health as fair, poor or very poor, i.e. who were in less than good health, were regarded as having ‘poor self-rated health’. Long sickness absence was defined by a total number of six or more self-reported days of absence from work for health reasons during the last 12 months.

Reports of no (score 0), minor (1) or major (2) limitations in climbing several flights of stairs (item 1) and in moderate activities such as moving a table, pushing a vacuum cleaner, bowling or playing golf (item 2) were used as an indicator of limited physical functioning (total score of 2 and more) and thus of physical health. The two items or questions about such limitations in daily physical activities were taken from the SF-12 Health Survey, a validated shorter form of the SF-36 Health Survey Questionnaire [37].

And finally, stress and burnout symptoms were used as mental health outcomes. Stress was assessed by a well-validated single-item measure of the general psychological stress symptoms used in the Occupational Stress Questionnaire [38]. Respondents were asked to indicate if and how strongly they currently felt stressed and experienced stress feelings or symptoms such as psychological tension, restlessness, nervousness, anxiety or sleeplessness due to a troubled mind, and to answer on a 5-point Likert scale varying from “not at all” (score 1) to “very much” (5). Responses of “much” (4) and “very much” (5) were combined and categorized as ‘strong stress feelings’. Burnout was measured by a selection of six items from the Copenhagen Burnout Inventory [39]. Three items each were taken from two 6- and 7-item subscales of the CBI used to measure personal burnout (e.g. “How often do you think: ‘I can’t take it anymore’?”) and work burnout (e.g. “Are you exhausted in the morning at the thought of another day at work?”). As industrial and construction workers usually do not work with clients, the third and client-related dimension of burnout was not applicable and therefore excluded. Response categories on a 5-point scale varied from “never” (score 0) to “always” (4). Since a principal component analysis of these six items revealed only one factor (with an eigenvalue greater than one) accounting for 56% of the total variance, a consolidated single measure was created and used with an accumulated score reaching a maximum of 24 and good internal consistency (Cronbach’s alpha = .84). A total score of 16 and more of this multiple-item measure was categorized as ‘increased burnout symptoms’.

### Statistical analyses

First, all the physical and psychosocial work factors considered were stratified by social status or class in order to explore if social gradients existed for all these factors, and which of them were negatively and which positively associated with social class. Subsequently, prevalence rates of health outcomes were calculated for the non-exposed (reference group) and exposed (at-risk group) study participants. The associations between all work factors and health outcomes were then tested using logistic regression analysis and sex- and age-adjusted odds ratios (aOR) were calculated in order to determine which of these adverse working conditions emerged as health risk factors as expected. And finally, different additional and stepwise multivariate logistic regression analyses were performed and multiple-adjusted OR were calculated to study the existence, dimensions and direction of the social gradients in health as well as the role of physical and psychosocial work factors in explaining this gradient. A first regression model included only social class as the independent variable and sex and age as control variables. In a second model, adverse working conditions that were negatively associated with social class were added as potential confounders or mediators. A third model included only adverse working conditions that were positively associated with social class and could possibly mask the social gradient in health. And in the fourth model, relationships between social class and health outcomes were adjusted for all job characteristics and work factors which had previously been shown to be detrimental to health in one respect or another. Additionally, possible interaction effects between social class and sex (and age) were tested in the basic and fully specified models. Statistical analyses were not stratified for both sexes due to the low number and proportion of women in the study sample (see Table 1). Instead, all associations or OR were controlled for sex (and age).

## Results

**Figure 1:**
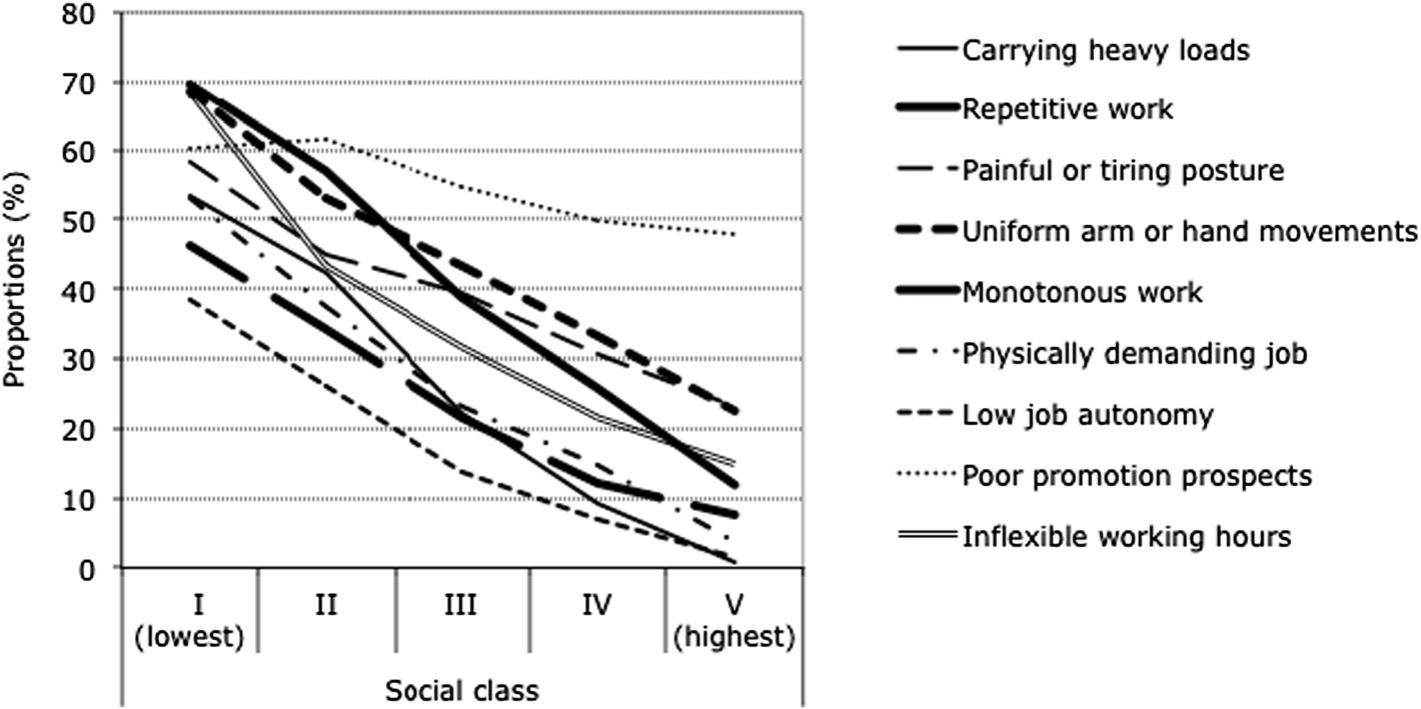
Adverse working conditions negatively associated with social class.

**Figure 2:**
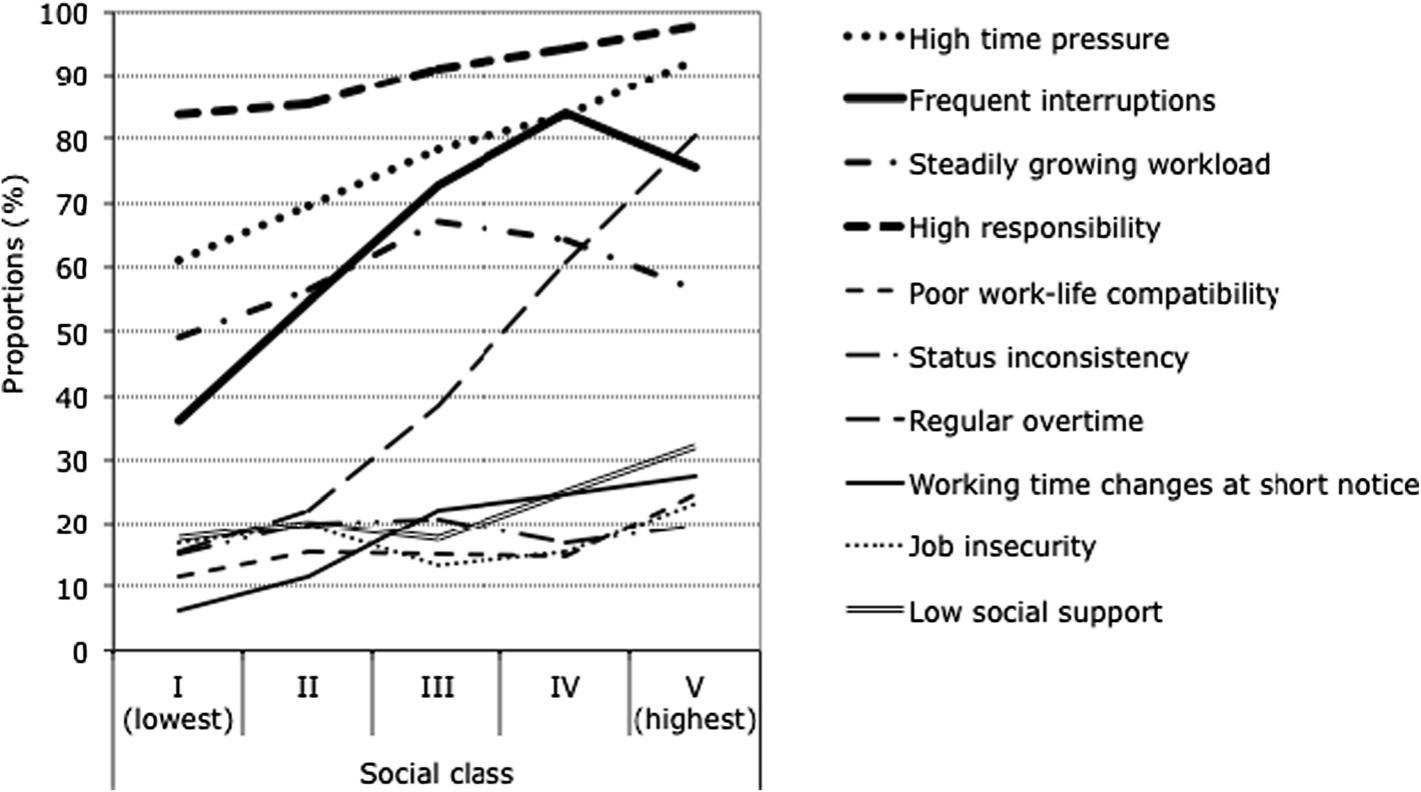
Adverse working conditions positively associated with social class.

Associations between social class and all studied working conditions almost consistently showed clear gradients. But while some of these adverse working conditions declined gradually and strongly in frequency with higher status, others rose steadily, as illustrated in Figures 1 and 2. Social gradients to the disadvantage of lower status groups or social classes were found, as usual, particularly for all physical work demands and exposures and for typical psychosocial characteristics of low-status occupations and blue-collar jobs, such as monotonous work, inflexible working hours or low job autonomy (see Figure 1). In contrast, reversed social gradients were found for other adverse psychosocial work factors that are characteristic of high-status occupations and white-collar jobs such as high time pressure, frequent interruptions, regular overtime or poor work-life compatibility (see Figure 2). Contrary to expectations, job resources such as job security, and social support at work (all recoded as a shortage or absence of such resources and hence as adverse working conditions) were not found to be less common in the lower classes, in fact rather the opposite.

Significant associations between various physical and psychosocial working conditions on the one hand and several general, physical and mental health outcomes on the other were found almost throughout and even after adjustment for sex and age and education (see Table 3). The strongest risk factors for poor self-rated health and limited physical functioning in daily activities were mainly physical work demands and some negative psychosocial work factors such as poor work-life compatibility, low social support, job insecurity and/or status inconsistency. An increased risk of long or frequent sickness absence (for six or more days per year) was particularly observed for employees having no flexible work time and experiencing monotony at work, poor work-life compatibility and job insecurity. Strong feelings of stress, and in particular increased symptoms of burnout and exhaustion, were found to be associated with almost all adverse working conditions but were most common under conditions of high psychosocial work demands and low job resources. Poor work-life compatibility and low social support turned out to be the strongest of all stressors and burnout correlates.

**Table 3:** Associations of various job characteristics and working conditions with different health outcomes

	**Poor self-rated health**		**Limited physical functioning (2–4)**		**Long sickness absence (6+ days/yr)**		**Strong stress feelings (4–5)**		**Increased burnout symptoms (16–24)**	
	**%**^**a**^	**aOR**^**b**^	**%**^**a**^	**aOR**^**b**^	**%**^**a**^	**aOR**^**b**^	**%**^**a**^	**aOR**^**b**^	**%**^**a**^	**aOR**^**b**^
**Total study population**	**14.1**		**9.2**		**15.5**		**15.1**		**6.4**	
**‘Blue-collar’ job characteristics**										
• Poor promotion prospects (57.2%^d^)	8.9	1	6.4	1	12.7	1	10.0	1	3.4	1
	17.6	2.16***	11.2	1.69**	17.3	1.44**	19.0	2.14***	8.6	2.79***
• Uniform arm or hand movements^c^ (49.7%^d^)	11.3	1	6.5	1	12.7	1	13.8	1	5.5	1
	16.9	1.46**	12.0	1.64**	18.4	1.26	16.3	1.32*	7.4	1.48
• Repetitive work^c^ (48.7%^d^)	10.4	1	5.1	1	12.2	1	14.3	1	5.2	1
	17.8	1.66***	13.4	2.30***	19.0	1.37*	15.8	1.29	7.8	1.97**
• Painful or tiring posture^c^ (43.5%^d^)	8.6	1	5.0	1	12.5	1	12.2	1	3.6	1
	21.3	2.71***	14.6	2.96***	19.2	1.46**	18.8	1.80***	10.2	3.59***
• Inflexible working hours (41.4%^d^)	12.3	1	7.4	1	12.0	1	14.7	1	5.8	1
	16.6	1.23	11.9	1.29	20.6	1.67**	15.7	1.24	7.4	1.68*
• Carrying heavy loads^c^ (33.4%^d^)	12.3	1	7.0	1	13.7	1	14.5	1	5.7	1
	17.6	1.41*	13.8	1.76***	18.9	1.19	16.0	1.29	8.0	1.96**
• Physically demanding job (32.5%^d^)	11.0	1	7.2	1	13.3	1	14.5	1	5.1	1
	20.5	1.94***	13.5	1.55*	19.8	1.36*	17.1	1.44*	9.4	2.73***
• Monotonous work^c^ (29.3%^d^)	11.2	1	6.4	1	12.3	1	13.4	1	4.4	1
	21.1	2.02***	16.2	2.57***	23.3	1.85***	19.2	1.73***	11.4	3.66***
• Low job autonomy (21.8%^d^)	12.7	1	7.7	1	14.0	1	14.4	1	5.7	1
	18.0	1.29	14.7	1.53*	20.2	1.37*	17.9	1.49*	9.3	2.29***
**‘White-collar’ job characteristics**										
• High responsibility (88.4%^d^)	14.8	1	8.7	1	20.5	1	11.6	1	6.7	1
	13.9	1.01	9.1	1.21	14.8	0.82	15.7	1.46	6.4	1.11
• High time pressure (73.3%^d^)	10.7	1	8.2	1	15.8	1	8.1	1	3.1	1
	15.2	1.70**	9.4	1.37	15.4	1.14	17.9	2.50***	7.7	2.54***
• Frequent interruptions (59.9%^d^)	14.8	1	10.4	1	17.2	1	9.3	1	3.0	1
	13.5	1.06	8.5	1.04	14.2	0.98	19.1	2.24***	8.6	2.88***
• Steadily growing workload (58.3%^d^)	14.8	1	8.3	1	14.8	1	8.7	1	3.0	1
	13.3	0.91	10.0	1.33	15.9	1.19	19.8	2.68***	8.9	3.29***
• Regular overtime (33.3%^d^)	13.7	1	10.4	1	16.8	1	12.1	1	4.8	1
	14.3	1.35	6.9	1.04	12.6	0.91	21.3	1.94***	9.6	1.94**
• Low social support (20.8%^d^)	12.1	1	8.8	1	14.8	1	11.0	1	3.6	1
	20.6	1.99***	10.6	1.34	17.2	1.26	29.4	3.24***	16.6	5.14***
• Status inconsistency (18.7%^d^)	12.6	1	8.6	1	15.1	1	13.8	1	5.1	1
	20.1	1.93***	10.6	1.51*	15.9	1.14	20.4	1.54**	12.4	2.54***
• Job insecurity (17.5%^d^)	12.3	1	9.0	1	13.6	1	13.5	1	5.3	1
	21.0	1.99***	9.7	1.23	22.8	1.97***	22.3	1.85***	11.9	2.46***
• Work time changes at short notice (15.3%^d^)	13.9	1	9.8	1	15.3	1	13.6	1	5.4	1
	14.6	1.24	6.8	1.04	15.2	1.20	23.7	1.88***	12.1	2.28***
• Poor work-life compatibility (15.2%^d^)	12.8	1	8.7	1	14.5	1	12.2	1	4.1	1
	20.9	2.05***	12.0	1.77**	20.8	1.82***	31.4	3.43***	19.9	6.64***

*p≤.05; **p<.01; ***p<.001.

^a^ Prevalence rates regarding health outcome of characterised group (lower percentage) and reference group (upper percentage).

^b^ OR adjusted for sex, age and education (with those not exposed and not working under such condition as reference group).

^c^ Applies partly, largely or fully to the job situation.

^d^ Frequency of job characteristic in the study sample.

Our main research question concerned the role and contribution of various working conditions to explaining the social inequalities in health (see Table 4): First, clear and partly very strong social gradients were observed for general and physical health outcomes (model I). These associations were found to be partly mediated and explained by typical blue-collar job characteristics and work factors such as physical work demands and low job autonomy and work time flexibility (model II). In contrast, controlling for white-collar job characteristics and work factors completely failed to explain the observed health inequalities with respect to social class, or even had a contrary effect and strengthened the social gradient (model III). Second, social gradients (even though showing fewer linear associations) were also found for the mental health outcomes studied such as psychological stress and burnout, but in the opposite direction (model I). The higher the social class, the higher did the relative risk of having strong stress feelings and reporting increased burnout symptoms tend to be. Unlike the observed social gradient in general and physical health, this reversed social gradient in mental health cannot be explained by blue-collar job characteristics and physical work factors (model II), but was found to be explained largely by white-collar job characteristics and psychosocial work factors (model III). Since Pearson’s correlation coefficients, as measures of association between all these working conditions, did not exceed values of .44, basically all working conditions were included in the multivariate analyses. After statistical adjustments had been made for all considered work factors (except for the item on high responsibility at work which did not show significant associations with health outcomes) and covariates, the observed gradients in both directions were substantially reduced and/or were no longer statistically significant (model IV).

**Table 4:** Social gradient in self-reported health outcomes and different white and blue-collar job characteristics and working conditions as possible explanatory factors

	**Poor self-rated health**		**Limited physical functioning (2–4)**		**Long sickness absence (6+ days/year)**		**Strong stress feelings (4–5)**		**Increased burnout symptoms (16–24)**	
	**%**	**aOR**	**%**	**aOR**	**%**	**aOR**	**%**	**aOR**	**%**	**aOR**
**Model I**^a^										
Social class										
I (lowest)	18.7	1	17.7	1	24.0	1	14.9	1	6.1	1
II	14.1	0.78	8.3	0.51***	16.2	0.61**	14.1	0.96	5.5	0.90
III	13.0	0.70	7.9	0.48**	13.5	0.51***	13.3	0.93	4.5	0.82
IV	10.1	0.54*	3.4	0.21***	9.7	0.34***	19.3	1.43	11.0	2.04*
V (highest)	8.2	0.43*	3.0	0.19***	4.5	0.15***	19.5	1.44	9.7	1.75*
Social class*age		0.98*		1.00		1.00		0.99*		1.00
Social class*sex (female)		0.99		1.32		1.55**		1.22		1.56*
**Model II**^b^										
Social class										
I (lowest)		1		1		1		1		1
II		1.00		0.64*		0.72		1.08		1.36
III		1.03		0.74		0.75		1.10		1.82
IV		0.85		0.29**		0.50*		1.99**		6.55***
V (highest)		0.78		0.43		0.26**		2.25**		7.37***
**Model III**^c^										
Social class										
I (lowest)		1		1		1		1		1
II		0.66*		0.45***		0.57**		0.65*		0.57
III		0.61*		0.43**		0.54**		0.58*		0.47
IV		0.42**		0.17***		0.35***		0.75		1.01
V (highest)		0.26***		0.16***		0.14***		0.61		0.64
**Model IV**^d^										
Social class										
I (lowest)		1		1		1		1		1
II		0.88		0.56*		0.64*		0.69		0.72
III		0.90		0.61		0.71		0.57*		0.73
IV		0.68		0.25**		0.46*		0.83		1.92
V (highest)		0.45		0.35		0.21***		0.72		1.46
Social class*age		0.99		0.99		1.00		0.98**		1.00
Social class*sex (female)		0.98		1.13		1.34**		1.10		1.30

*p ≤ .05; **p < .01; ***p < .001.

^a^ OR adjusted for sex and age.

^b^ OR additionally adjusted for blue-collar job characteristics covering mainly physical work factors.

^c^ OR additionally adjusted for white-collar job characteristics covering mainly psychosocial work factors.

^d^ OR additionally adjusted for blue AND white-collar job characteristics, i.e. for physical AND psychosocial work factors.

Possible interactions with social class were tested for sex and age in models I and IV, and isolated effects were found, although not consistently, across all health outcomes as shown in Table 4: Social gradients or health inequalities declined slightly with increasing age, but only for self-rated health and feelings of stress. And health inequalities tended to be more pronounced in women than men, except for self-rated health.

## Discussion

The main aim of this study was to explore the partly understudied and presumably inconsistent contribution of the work environment to explaining health inequalities. In this context and even more in an industrial work environment, the physical and psychosocial working conditions had barely been studied together previously and in such quantity, breadth and variety. Health inequalities were explored with regard to educational level and occupational position as the two indicators of social status available in this study, and with regard to five self-reported general, physical and mental health outcomes that have to our knowledge never before been studied simultaneously in this respect.

The main findings of our study can be summarised as follows: First, nearly all work factors considered showed graded associations with social class, or rather with combined educational and occupational status. Physical work demands and other typical blue-collar job characteristics such as monotony and low autonomy at work or low flexibility of working hours were found to be negatively associated with social status and much more frequent among the lower classes, whereas psychosocial work demands and typical white-collar job characteristics such as high time pressure, regular overtime, frequent interruptions or poor work-life compatibility were found to be positively related to social status and to be more common among the higher classes. This is fully consistent with expectations, since poorly qualified workers usually have low-status manual and physically demanding jobs with low discretion whereas highly qualified workers mostly have high-status non-manual and more intensive and psychosocially demanding jobs.

However, and against expectations, lower-class workers in our study sample were not more likely to report job insecurity, or low social support at work. In an industrial work environment offering many blue-collar jobs, a comparatively poor qualification or education and low occupational position may be less disadvantageous and thus less related to job insecurity that is the case in the service sector involving a majority of white-collar jobs. In any case, this study result was consistent with findings and reports from previous studies, which found social support at work or job security not to be clearly related to social or occupational class [10,21] or to socioeconomic or educational status [11,25].

Second, all adverse working conditions with the sole exception of high responsibility at work turned out to be health risk factors, even after controlling for education and other covariates. These results were basically in line with expectations and with findings from other studies [inter alia, 31]. But while only some of these working conditions were shown to increase the risk for poor general and physical health outcomes, nearly all of them proved to be strong risk factors for mental health disorders, and particularly for burnout symptoms. This might be explained by the strong fatigue component of the burnout measure, which includes items relating to physical and emotional exhaustion.

Third, social inequalities or more precisely social gradients were found for all health outcomes but did not run in the same direction. Health disadvantages of the lower classes were observed only for general and physical health outcomes (self-rated health, limited physical functioning, sickness absence), but were not found for mental health outcomes (psychological stress, burnout symptoms) – indeed, quite the contrary. These health inequalities in both directions, to the disadvantage of both the lower and higher classes, were significantly reduced after adjustment for all considered work factors. But while blue-collar job characteristics, and mainly physical work factors, largely or at least partly explained the observed social inequalities in general and physical health outcomes, they did not contribute to the reversed social gradient found for mental health outcomes. The very reverse was the case for white-collar job characteristics, and predominantly psychosocial work factors, which did not make any contribution to explaining the gradient in general and physical health, but were mainly responsible for the social inequalities in mental health. Even more, adjusting for white-collar job characteristics tended to increase rather than reduce the social gradient in general and physical health. And adjusting for blue-collar job characteristics likewise increased the reversed social gradient in mental health.

The study results regarding the different role of physical and psychosocial work factors in explaining widely observed health inequalities support some previous findings and are partly in line with earlier studies that found:

• inconsistent mediating effects of psychosocial work resources and demands on the education-health relationship [25],

• psychosocial working conditions to have only minor or contrasting effects on health inequalities [26],

• wider health inequalities when considering psychosocial working conditions [19],

• that the relationship between social class and health was reinforced when controlling for psychosocial job demands [24],

• that ergonomic and physical exposures make a significant contribution but psychological work demands do not contribute to inequalities in health [15], and

• that (psychological) job demands and social support at work do not contribute to explaining the association between occupational class and perceived general health [21].

Our finding of a reversed social gradient in mental health is inconsistent with the widely documented social inequalities in physical and general health and illness, and similar social gradients found for severe mental disorders [40,41], and is particularly contrary to the quite recently published study of Qiu et al. [25] in which higher education was found to be associated with lower levels of perceived stress and self-reported depression. However, this rather unexpected result of stronger stress feelings and more frequent burnout symptoms among the upper classes may be interpreted as a complementary rather than a contradictory finding in a previously unexplored or understudied work environment and study population, and it may be attributable to different and particularly higher psychosocial job demands and stressors and lower job resources and rewards for higher educated employees in the industry sector compared to the commonly explored service sector.

Moreover, findings of earlier studies have been much less consistent for minor mental health problems than for severe mental disorders as some studies found only weak or partly reverse associations and gradients for common mental disorders based on conventional indicators or measures of social or socioeconomic status [40,42]. Such inconsistent results may be related to the measure used as some studies suggest which have examined multiple measures of social status or socioeconomic position simultaneously [40,42]. Such measures were shown to be differently associated with (mental) health and interrelated with each other and with regard to various health outcomes, and to be partially independent and partially inter-dependent determinants of health [40,43-45].

### Strengths and limitations of the study

The present study was based on a survey carried out in the secondary sector where the full spectrum of physical and psychosocial work environments can be found. The study makes a significant contribution to the research by including a total of nineteen working conditions and five very different health outcomes. No prior study of this research topic has covered and explored such a broad range of job characteristics and work factors (from physical workloads, exposures and strains to psychosocial work demands, pressures and resources) and so many diverse general, physical and mental health outcomes simultaneously. Moreover, the study provided evidence from a heterogeneous sample that was not biased in favour of middle-class members and primarily focused on white-collar workers, high school graduates or civil servants as is usual and was seen in several recent studies [17,19,24,30,31]. The study sample covered the full range of social status groups, from unskilled industrial and construction workers who do mainly manual or physical work to highly skilled non-manual workers who perform professional, administrative or managerial work. In contrast to other population studies and their samples with only small proportions of low-status individuals [17,30], our study population included a comparably high proportion (23%) of unskilled blue-collar workers. In addition, by combining educational level with its strongly associated occupational position and identifying five different social status groups, we used a more valid and appropriate measure for social status or class than many previous studies. Given the multidimensional nature of social status or socioeconomic position [40] and the existence of interrelationships between different measures of social or socioeconomic status and health [44,45] the use of such a combined two-item measure seems to be a more valid and appropriate way of assessing social status or class than only using either educational level or occupational position. And finally, instead of differentiating between physical and psychosocial work factors as usual we distinguished typical blue-collar and primarily white-collar job characteristics (which have all been found to be health-related risk factors and were either positively or negatively associated with social class) from each other, and their potential effects on health inequalities were analysed both simultaneously *and* separately. This approach was essential to explore possible contrasting or counteracting effects of such diverse working conditions on health inequalities.

In light of the study’s cross-sectional design, causal interpretations cannot be drawn. Since the participating companies were not randomly selected and their workforces do not represent the entire secondary sector or even individual industries, the study results may not be generalizable to other populations, companies or industries. Neither can a selection bias be excluded in light of the rather low response rates among the workforces of the participating companies. And since we used self-reported data, a reporting or misclassification bias may also have occurred. However, there are no specific reasons or indications to assume that our results are systematically biased, overstated or not transferable to other populations. In addition, plausibility of the findings and their consistency across different companies or workforces (not shown) and with earlier studies, fairly strong associations and clear gradients, the large heterogeneity of the study sample and comprehensible reasons for the low return rates rather speak against such interpretations and do not suggest a limited scope, validity and generalizability of the study findings.

Specific reasons for the rather low return or response rates (below 50%) in two of the four participating companies included language difficulties and understanding problems among foreign employees and a broad uncertainty and displeasure as a result of ongoing restructuring and redundancies in one of the companies. These factors may have resulted in self-exclusion by dissatisfied and poorly qualified blue-collar workers, leading to a systematic selection bias towards a ‘healthy and happy worker effect’, and thus to an under-estimation of health inequalities and/or specific work-related risk factors such as job insecurity or low job autonomy. However, this would mean that the social gradients in health and negative health effects of some adverse work factors could effectively be stronger than assumed and observed in this study.

To take another example, education, occupational position and income may be not interchangeable measures of social status due to the less structured social stratification and increased status inconsistency broadly observed over the past decades. However, previous studies that used different indicators of social status or socioeconomic position based either on education [25], income [15], occupation [16] or occupational class [19,21,24,26,46], on multiple measures [27] or a combined social classification similar to that used in this study [10] yielded partly consistent findings as regards the role and contribution of working conditions to health inequalities. They consequently give no strong reason to assume that the use of other indicators or single-item measures of social status would have made much difference in this study. Moreover, studies examining multiple measures of social or socioeconomic status repeatedly showed clear gradients and strong and consistent associations with health [43-45,47].

After all, the main study results support findings from previous and similar studies [25]. Even the unexpected and inconsistent finding of a reversed social gradient in mental health outcomes is at least partly in line with findings from earlier studies [40,42].

## Conclusion

We have shown in our study that the various physical and psychosocial working conditions that were studied contributed substantially to the health inequalities found in the sample. We also demonstrated the importance of a differential consideration of blue- and white-collar job characteristics in explaining the social gradient in health. The study results clearly indicate that the traditionally observed social gradient in health to the disadvantage of blue-collar workers may be partly masked and would have been even more accentuated without the comparatively high psychosocial work demands and partly limited resources that are characteristic of white-collar jobs. These white-collar job characteristics in turn were found to be mainly responsible for increased stress feelings and burnout symptoms among the higher social classes and the reversed social gradient in mental health to the disadvantage of white-collar workers shown in this study. Our findings suggest that at least in the industry sector, the work environment not only puts blue-collar workers in low-status manual jobs and their (physical and general) health at risk but is also detrimental to the (mental) health of white-collar workers in high-status and non-manual jobs, and may be co-responsible for increasingly prevalent mental health problems and particularly increased stress and burnout symptoms observed among the workforce in Switzerland [48] and many other countries. These results, of course, first need to be confirmed and supported by future studies among other working populations before conclusions can be drawn and practical implications aiming to reduce health inequalities derived. However, our study results point to possible changes in the future regarding health inequalities and its affected populations. It will have to be determined whether such a reversed social gradient in mental health can also be observed in other and broader sections of the working population. If so, this would have strong implications for the future, particularly as it can be expected that both the number of white-collar workers and the importance of the psychosocial work environment will further grow for two reasons: Firstly, in the course of increasing tertiarisation of the economy, industrial and therefore physical and manual work is on the decline whereas service and non-manual work is on the rise. Secondly, and against the background of an ongoing intensification of work, a gradual transition can be observed in many industries to more flexible and precarious working arrangements and employment contracts and, as a result, increasing psychosocial work demands (e.g. long hours, high time pressure, low schedule flexibility, job insecurity) and increasing difficulties in combining work and private life.

## Competing interests

The authors declare that they have no competing interests.

## Authors’ contributions

OH designed and conducted the study, collected the data, prepared and revised the manuscript and performed the statistical analyses. GB contributed to the conception and design of the study and participated in the development and construction of the questionnaire. Both authors approved the final manuscript.

## Pre-publication history

The pre-publication history for this paper can be accessed here:

http://www.biomedcentral.com/1471-2458/13/1170/prepub
